# Supplementary material for: Heparin-based hydrogel scaffolding alters the transcriptomic profile and increases the chemoresistance of MDA-MB-231 triple-negative breast cancer cells
Source: Biomater Sci. 2020 Feb 13;8(10):2786–96. doi: 10.1039/c9bm01481k (PMC7497406; doi:10.1039/c9bm01481k)
Supplement: Supplementary file 2 [file BM-008-C9BM01481K-s002.zip › Supplementary File 4/HP-BhydrogelvControl/Pathways/my_analysis.Gsea.1545201079907/HALLMARK_COAGULATION.html]

Details for gene set HALLMARK\_COAGULATION[GSEA]

|  || Dataset | expr.class.cls#HYD\_versus\_CONTROL.class.cls#HYD\_versus\_CONTROL\_repos |
| Phenotype | class.cls#HYD\_versus\_CONTROL\_repos |
| Upregulated in class | CONTROL |
| GeneSet | HALLMARK\_COAGULATION |
| Enrichment Score (ES) | -0.38736016 |
| Normalized Enrichment Score (NES) | -1.6547214 |
| Nominal p-value | 0.0033898305 |
| FDR q-value | 0.008211597 |
| FWER p-Value | 0.03 |
Table: GSEA Results Summary

  

Fig 1: Enrichment plot: HALLMARK\_COAGULATION      
 Profile of the Running ES Score & Positions of GeneSet Members on the Rank Ordered List

  

| PROBE | DESCRIPTION (from dataset) | GENE SYMBOL | GENE\_TITLE | RANK IN GENE LIST | RANK METRIC SCORE | RUNNING ES | CORE ENRICHMENT || 1 | MAFF | na |  |  | 475 | 2.376 | 0.0114 | No |
| 2 | RAPGEF3 | na |  |  | 1047 | 1.841 | 0.0096 | No |
| 3 | CTSK | na |  |  | 1864 | 1.420 | -0.0114 | No |
| 4 | SIRT2 | na |  |  | 2749 | 1.102 | -0.0408 | No |
| 5 | MASP2 | na |  |  | 2753 | 1.101 | -0.0242 | No |
| 6 | SH2B2 | na |  |  | 2821 | 1.080 | -0.0112 | No |
| 7 | FURIN | na |  |  | 3731 | 0.863 | -0.0456 | No |
| 8 | HTRA1 | na |  |  | 3847 | 0.842 | -0.0388 | No |
| 9 | MMP15 | na |  |  | 4121 | 0.786 | -0.0411 | No |
| 10 | GP1BA | na |  |  | 4183 | 0.773 | -0.0324 | No |
| 11 | MST1 | na |  |  | 4350 | 0.742 | -0.0298 | No |
| 12 | RGN | na |  |  | 4430 | 0.727 | -0.0228 | No |
| 13 | SERPINB2 | na |  |  | 4996 | 0.627 | -0.0428 | No |
| 14 | FYN | na |  |  | 5004 | 0.625 | -0.0337 | No |
| 15 | TMPRSS6 | na |  |  | 5664 | 0.530 | -0.0601 | No |
| 16 | BMP1 | na |  |  | 5841 | 0.508 | -0.0615 | No |
| 17 | LRP1 | na |  |  | 6291 | 0.446 | -0.0782 | No |
| 18 | CASP9 | na |  |  | 6333 | 0.439 | -0.0737 | No |
| 19 | S100A1 | na |  |  | 6693 | 0.394 | -0.0864 | No |
| 20 | C3 | na |  |  | 7510 | 0.306 | -0.1245 | No |
| 21 | FBN1 | na |  |  | 8665 | 0.184 | -0.1820 | No |
| 22 | C1S | na |  |  | 8670 | 0.184 | -0.1794 | No |
| 23 | F3 | na |  |  | 9090 | 0.142 | -0.1992 | No |
| 24 | C2 | na |  |  | 9829 | 0.060 | -0.2369 | No |
| 25 | CTSH | na |  |  | 9951 | 0.046 | -0.2425 | No |
| 26 | CFB | na |  |  | 10199 | 0.015 | -0.2552 | No |
| 27 | GNB2 | na |  |  | 10378 | 0.009 | -0.2644 | No |
| 28 | THBD | na |  |  | 10820 | -0.042 | -0.2868 | No |
| 29 | CRIP2 | na |  |  | 11332 | -0.093 | -0.3121 | No |
| 30 | PLAU | na |  |  | 11513 | -0.113 | -0.3198 | No |
| 31 | USP11 | na |  |  | 11584 | -0.124 | -0.3216 | No |
| 32 | PEF1 | na |  |  | 11755 | -0.146 | -0.3283 | No |
| 33 | MMP3 | na |  |  | 11975 | -0.170 | -0.3371 | No |
| 34 | C1R | na |  |  | 12054 | -0.180 | -0.3385 | No |
| 35 | WDR1 | na |  |  | 12130 | -0.190 | -0.3395 | No |
| 36 | APOC1 | na |  |  | 12152 | -0.193 | -0.3376 | No |
| 37 | F8 | na |  |  | 12313 | -0.216 | -0.3427 | No |
| 38 | CTSE | na |  |  | 12424 | -0.222 | -0.3451 | No |
| 39 | DPP4 | na |  |  | 12540 | -0.229 | -0.3476 | No |
| 40 | CAPN5 | na |  |  | 12728 | -0.247 | -0.3536 | No |
| 41 | CFH | na |  |  | 12948 | -0.278 | -0.3608 | No |
| 42 | PECAM1 | na |  |  | 13230 | -0.314 | -0.3707 | No |
| 43 | TIMP3 | na |  |  | 13429 | -0.342 | -0.3759 | No |
| 44 | PDGFB | na |  |  | 13460 | -0.347 | -0.3721 | No |
| 45 | KLF7 | na |  |  | 13490 | -0.352 | -0.3683 | No |
| 46 | ANG | na |  |  | 13761 | -0.397 | -0.3764 | No |
| 47 | PLAT | na |  |  | 13867 | -0.414 | -0.3755 | No |
| 48 | PLG | na |  |  | 14094 | -0.429 | -0.3808 | Yes |
| 49 | SERPINA1 | na |  |  | 14126 | -0.430 | -0.3759 | Yes |
| 50 | CAPN2 | na |  |  | 14193 | -0.440 | -0.3726 | Yes |
| 51 | MMP11 | na |  |  | 14247 | -0.452 | -0.3685 | Yes |
| 52 | MMP1 | na |  |  | 14272 | -0.457 | -0.3628 | Yes |
| 53 | F2RL2 | na |  |  | 14504 | -0.491 | -0.3673 | Yes |
| 54 | CLU | na |  |  | 14511 | -0.492 | -0.3601 | Yes |
| 55 | TIMP1 | na |  |  | 14560 | -0.499 | -0.3550 | Yes |
| 56 | A2M | na |  |  | 15146 | -0.588 | -0.3767 | Yes |
| 57 | LGMN | na |  |  | 15311 | -0.619 | -0.3758 | Yes |
| 58 | RABIF | na |  |  | 15405 | -0.635 | -0.3710 | Yes |
| 59 | ACOX2 | na |  |  | 15508 | -0.652 | -0.3664 | Yes |
| 60 | GSN | na |  |  | 15576 | -0.665 | -0.3597 | Yes |
| 61 | CTSB | na |  |  | 16096 | -0.769 | -0.3752 | Yes |
| 62 | GNG12 | na |  |  | 16219 | -0.792 | -0.3695 | Yes |
| 63 | ISCU | na |  |  | 16260 | -0.800 | -0.3593 | Yes |
| 64 | ITGB3 | na |  |  | 16433 | -0.839 | -0.3555 | Yes |
| 65 | ADAM9 | na |  |  | 16444 | -0.841 | -0.3432 | Yes |
| 66 | TFPI2 | na |  |  | 16544 | -0.865 | -0.3352 | Yes |
| 67 | CSRP1 | na |  |  | 16883 | -0.950 | -0.3384 | Yes |
| 68 | ARF4 | na |  |  | 16900 | -0.956 | -0.3246 | Yes |
| 69 | ANXA1 | na |  |  | 16924 | -0.963 | -0.3111 | Yes |
| 70 | F12 | na |  |  | 16939 | -0.966 | -0.2971 | Yes |
| 71 | SPARC | na |  |  | 17223 | -1.058 | -0.2958 | Yes |
| 72 | APOA1 | na |  |  | 17228 | -1.058 | -0.2798 | Yes |
| 73 | ITGA2 | na |  |  | 17270 | -1.074 | -0.2656 | Yes |
| 74 | CFD | na |  |  | 17568 | -1.184 | -0.2630 | Yes |
| 75 | CFI | na |  |  | 17692 | -1.240 | -0.2506 | Yes |
| 76 | PREP | na |  |  | 17813 | -1.292 | -0.2371 | Yes |
| 77 | DUSP14 | na |  |  | 17869 | -1.318 | -0.2199 | Yes |
| 78 | S100A13 | na |  |  | 18233 | -1.519 | -0.2157 | Yes |
| 79 | DUSP6 | na |  |  | 18261 | -1.536 | -0.1937 | Yes |
| 80 | CTSO | na |  |  | 18318 | -1.582 | -0.1724 | Yes |
| 81 | LTA4H | na |  |  | 18589 | -1.771 | -0.1595 | Yes |
| 82 | RAC1 | na |  |  | 18642 | -1.836 | -0.1342 | Yes |
| 83 | PROS1 | na |  |  | 18766 | -1.970 | -0.1106 | Yes |
| 84 | PRSS23 | na |  |  | 18840 | -2.072 | -0.0828 | Yes |
| 85 | MSRB2 | na |  |  | 18841 | -2.074 | -0.0511 | Yes |
| 86 | CD9 | na |  |  | 18879 | -2.138 | -0.0204 | Yes |
| 87 | LAMP2 | na |  |  | 19026 | -2.430 | 0.0090 | Yes |
Table: GSEA details [plain text format]

  

Fig 2: HALLMARK\_COAGULATION      
 Blue-Pink O' Gram in the Space of the Analyzed GeneSet

  

Fig 3: HALLMARK\_COAGULATION: Random ES distribution      
 Gene set null distribution of ES for **HALLMARK\_COAGULATION**
